# Roles of Ubiquitination and SUMOylation on Prostate Cancer: Mechanisms and Clinical Implications

**DOI:** 10.3390/ijms16034560

**Published:** 2015-02-27

**Authors:** Zhenbang Chen, Wenfu Lu

**Affiliations:** Department of Biochemistry and Cancer Biology, Meharry Medical College, Nashville, TN 37208, USA; E-Mail: wlu@mmc.edu

**Keywords:** posttranslational modifications, ubiquitination, SUMOylation, prostate cancer

## Abstract

The initiation and progression of human prostate cancer are highly associated with aberrant dysregulations of tumor suppressors and proto-oncogenes. Despite that deletions and mutations of tumor suppressors and aberrant elevations of oncogenes at the genetic level are reported to cause cancers, emerging evidence has revealed that cancer progression is largely regulated by posttranslational modifications (PTMs) and epigenetic alterations. PTMs play critical roles in gene regulation, cellular functions, tissue development, diseases, malignant progression and drug resistance. Recent discoveries demonstrate that ubiquitination and SUMOylation are complicated but highly-regulated PTMs, and make essential contributions to diseases and cancers by regulation of key factors and signaling pathways. Ubiquitination and SUMOylation pathways can be differentially modulated under various stimuli or stresses in order to produce the sustained oncogenic potentials. In this review, we discuss some new insights about molecular mechanisms on ubiquitination and SUMOylation, their associations with diseases, oncogenic impact on prostate cancer (PCa) and clinical implications for PCa treatment.

## 1. Molecular Mechanisms of Ubiquitination and SUMOylation

Posttranslational modifications (PTMs) of proteins include enzymatic changes such as the addition of chemical group adducts such as ubiquitin (Ub), small ubiquitin-like modifiers (SUMO), phosphor, acetyl and methyl moieties [1,2]. PTMs play essential roles in gene regulation, cellular function, tissue development, and metabolism, whose alterations trigger cancers and other diseases. Unlike the processes of phosphorylation, acetylation, and methylation, the pathways of ubiquitination and SUMOylation are highly regulated and reversible processes that are normally involved in several enzymatic steps by multiple co-factors. On the other hand, the status of phosphorylation, acetylation, and methylation on substrate proteins may determine whether and how ubiquitin and SUMO moieties are conjugated to proteins in any given environments. More importantly, understanding how these distinct and influential processes impact protein homeostasis and other signaling pathways may provide valuable information that can lead to drug development for treating cancers and diseases [3,4,5,6,7].

### 1.1. Ubiquitination

**Figure 1:**
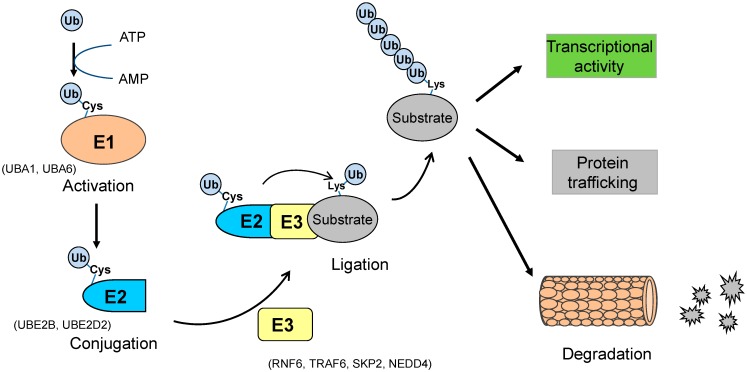
The ubiquitination pathway. Ubiquitin (Ub) protein moiety is activated by Ub-activating enzyme E1 (such as UBA1, UBA6) through the cysteine (Cys) residue of E1. Ub at E1 is transferred to the Cys residue of Ub-conjugating enzyme E2 (such as UBE2B, UBE2D2). Ub conjugated with E2 is catalytically transferred to the lysine (Lys) residue of a substrate protein by an E3 Ub ligase (such as RNF6, TRAF6, SKP2, NEDD4). Ubiquitinated proteins with the poly- (or mono-) ubiquitination linkage are subjected to proteasome-dependent degradation, trafficking between subcellular compartments, or the regulation of transcriptional activities.

Ubiquitination is the biochemical pathway in which a small protein called ubiquitin (Ub) is enzymatically linked to a substrate protein [8]. Ub is an evolutionally conserved protein (8.5 kDa) consisting of 76 amino acids with abundant distributions in eukaryotic cells [9,10,11]. Ub contains seven lysine residues K6, K11, K27, K29, K33, K48, and K63 through which the ubiquitination chain extends. Upon ubiquitination, the last amino acid (glycine 76) of Ub is covalently ligated to a lysine residue of the substrate protein through an isopeptide linkage between the *C*-terminal glycine of Ub and the amino group of lysine [12]. For low lysine-content proteins such as alternative read frame (ARF) with only one lysine, polyubiquitination may take place naturally in a lysine-independent manner. Substrate proteins can be modified by a single Ub moiety (termed as mono-ubiquitination) or poly-Ub chain (poly-ubiquitination) through the isopeptide linkage between 2 Ub moieties. Monoubiquitination may occur in a multiple manner if substrate proteins comprise several lysine residues. The monoubiquitination procedure is certainly required prior to the polyubiquitination that frequently occurs through a linkage between 2 Ub molecules at its seven lysines. Monoubiquitination plays a predominant role on protein trafficking, while polyubiquitination contributes to protein trafficking (through K63) and degradation (through K48). The ubiquitination procedure is accomplished in three enzymatic steps: Ub-activation, Ub-conjugation and Ub-ligation (Figure 1). Specifically, (a) Ub-activation: Ub is activated by E1, an Ub-activating enzyme (UBA1, UBA6), in an ATP-dependent manner. In this process, E1 binds Ub to catalyze the acyl-adenylation of the *C* terminus of Ub, and Ub is transferred to the cysteine residue of E1; (b) Ub-conjugation: Ub is conjugated with E2, the Ub-conjugating enzymes (UBE2B, UBE2D2). In this reaction, E2 physically interacts with both Ub and E1 to transfer Ub from E1 to the active site cysteine of E2; (c) Ub-ligation: Ub is transferred to the substrate by an E3 Ub ligase (RNF6, TRAF6, SKP2, and Nedd4), through an isopeptide linkage between substrate proteins and Ub moieties. In this reaction, the E3 enzyme interacts with both the substrate protein and the E2~Ub thioester to catalyze Ub transfer in the ubiquitination process. An E3 enzyme recognizes its substrate protein to mark it for protein trafficking among subcellular compartments in cellular functions (K63) or to designate it as a target of degradation through the proteasome (K48), depending on the lysine linkage of ubiquitination. Several hundreds of E3 Ub ligases have been reported in eukaryotes, and the linkages of ubiquitination are determined by the functional specificities of the E3 Ub enzymes. There are three subfamilies of E3 Ub enzymes: the homology to E6-AP carboxyl terminus (HECT), the really interesting new gene (RING) and U-box. The HECT domain of E3 enzymes first binds Ub at the active site of E3, and then the RING domain catalyzes the Ub transfer from the E2 enzyme to the substrate protein. RING domain-containing E3s (such as MDM2, SKP2) consist of more than 600 members, while HECT-type E3s (such as ARF-BP1, NEDD4) only have 28 members. Cullin-RING ligases (CRLs) are the major group of RING-type E3 ligases to mediate proteasomal degradation. Recently, U-box type Ub ligases (such as UFD2a) are termed as E4 Ub ligases, a new class of ubiquitination enzymes [13]. Given the various forms of ubiquitination linkages, the ubiquitination elongation of substrate proteins may be mainly recognized and determined by E3 Ub ligases through the structural determinants, including primary sequence, PTMs and protein folding state. Ubiquitin-interacting Motif (UIM) plays a critical role in the ubiquitination pathway for 26S-dependent proteasomal degradation and non-proteolytic protein trafficking. Ub molecules are recycled through the deubiquitination process, particularly for ubiquitinated proteins in non-proteolysis. Deubiquitination is the reverse process of ubiquitination to cleave Ub from the substrate protein, and is normally catalyzed by deubiquitination enzymes (DUB) or ubiquitin specific proteases (USPs) [14].

### 1.2. SUMOylation

**Figure 2:**
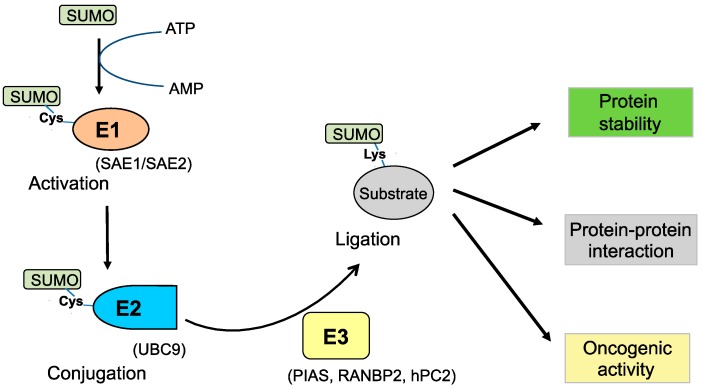
The SUMOylation pathway. The SUMO protein moiety is activated by SUMO-activating enzyme E1 (such as SAE1, SAE2) through the cysteine (Cys) residue of E1. SUMO at E1 is transferred to the Cys residue of SUMO-conjugating enzyme E2 (such as UBC9). SUMO linked to UBC9 is catalytically transferred to the lysine (Lys) residue of a substrate protein by a SUMO E3 ligase (such as PIAS, RANBP2, hPC2). Proteins modified with SUMOylation can play a critical role in oncogenic activity, protein–protein interactions, protein stability, or the regulation of transcriptional activities.

SUMOylation refers to the biochemical procedure in which a small ubiquitin-like modifier (SUMO) protein moiety is enzymatically conjugated to a substrate protein [15,16]. As an important machinery of PTMs in cells, SUMOylation also plays an essential role in the regulation of cellular processes and functions such as gene regulation, cell differentiation, tissue development and disease progression [17]. SUMO proteins (~12 kDa) contain about 100 amino acids, and have four isoforms (SUMO-1, SUMO-2, SUMO-3 and SUMO-4) [18,19]. Similar to the ubiquitination pathway, the SUMOylation procedure normally consists of three enzymatic steps: (a) SUMO-activation by enzyme E1 (SAE1/2); (b) SUMO-conjugation by enzyme E2 (UBC9); and (c) SUMO-ligation by enzyme E3 (PIAS/RANBP2) (Figure 2). SUMO is bound to its target protein via an isopeptide bond formed between the *C* terminal carboxyl group on the SUMO moiety and the amino group on the lysine residue of the substrate protein. Although SUMOylation has been reported in many proteins, SUMO modification is much less commonly detected for most proteins in cells as compared to Ub modification. The major reason for this difference is that SUMO modification does not directly target substrates for degradation [20]. The SUMOylation of substrate proteins is dependent not only on the consensus motif of SUMOylation but also on the growth microenvironments and stress stimuli. The SUMOylation possibility increases if the substrate proteins contain the consensus motif ΨKXE (where Ψ represents a large hydrophobic amino acid, and X represents any amino acid). It should be noted, however, that some reports show a large number of proteins (~40%) can be SUMOylated without the presence of the consensus sequence in the ΨKXE-binding pocket [21]. The SUMOylation process can be reversed by deSUMOylation to recycle SUMO molecules. DeSUMOylation is normally catalyzed by SUMO specific protease (SENP) that cleaves the SUMO group from SUMOylated proteins [22].

## 2. Ubiquitination and SUMOylation in Prostate Cancer

Ubiquitination and SUMOylation pathways, together with deubiquitination and deSUMOylation reversing steps, are tightly regulated to maintain a balanced homeostasis on proteins in normal cells. In addition, recent studies have revealed that dysregulation of ubiquitination and SUMOylation contributes to the initiation and progression of various human cancers including prostate cancer (PCa) by directly attenuating protein functions of tumor suppressors and/or potentiating the hyperactive roles of oncogenes. Several essential signaling pathways are aberrantly regulated by ubiquitination and SUMOylation modifications in cancer cells through affecting the binding abilities and levels of some key co-factors. Altered forms of ubiquitination and/or SUMOylation cause an abnormal accumulation of oncoproteins and transcriptional factors in subcellular compartments, which drives cancer progression through regulation of gene expression and cell reprogramming. Recent studies report that the ubiquitination pathway can work in concert with the SUMOylation to modulate cellular functions when these two modifications target the same substrate protein, and the modification preference may generate influential oncogenic impacts in cells. Both ubiquitination and SUMOylation make important contributions to the regulation of key tumor suppressors, oncogenes and the associated signaling pathways in PCa.

### 2.1. Ubiquitination in Prostate Cancer

**Figure 3:**
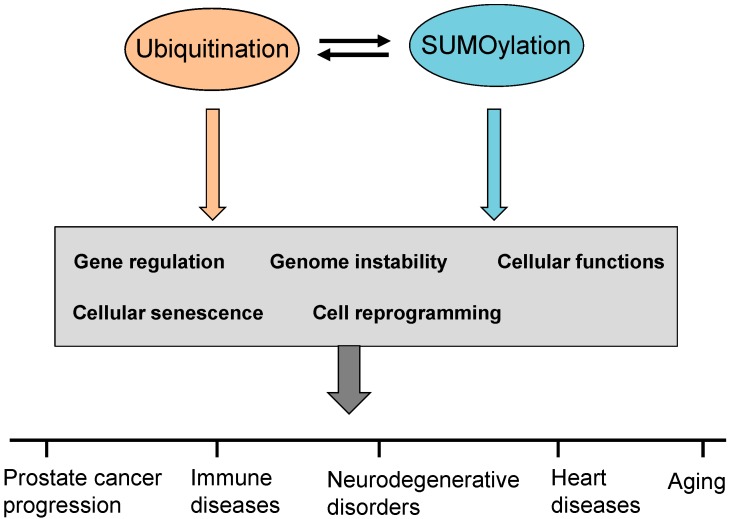
Ubiquitination and SUMOylation for prostate cancer and diseases. Aberrant alterations of relevant catalytic enzymes and oncoproteins upon ubiquitination/SUMOylation are involved in gene regulation, genome instability, cellular functions, cellular senescence and cell reprogramming *etc.* Dysregulation of ubiquitination and SUMOylation pathways contributes to the development of prostate cancer and other diseases.

Emerging evidence revealed that aberrant alterations of enzymes in the ubiquitination pathway directly promote the initiation and progression of various human cancers. Therefore, targeting ubiquitin enzyme activities at all three steps may be a potential and novel chemotherapeutic approach for cancer control (Figure 3). Knockdown of Ub-activating enzyme E1 results in apoptosis of leukemia and multiple myeloma cells, suggesting the potential of E1 inhibition for the treatment of leukemia and multiple myeloma [23]. The human Ub-conjugating enzymes (E2), UBE2C and UBE2S, cooperate with E3 Ub ligase anaphase-promoting complex/cyclosome (APC/C) to regulate the cell cycle in cancers. UBE2C elevation is observed in lung, bladder and ovarian cancers, underscoring its potential as a cancer biomarker. The E2 enzyme UBE2B, increases the polyubiquitination of β-Catenin, a protein that is frequently upregulated in cancer cells [24]. In addition, UBE2B cooperates with MDM2, an E3 Ub ligase, in a complex to assist the ubiquitination and degradation of the p53 tumor suppressor [25].

The ubiquitination pathway is essential to maintain protein function, protein trafficking and protein homeostasis of tumor suppressors and oncogenes in various human cancers including PCa. Inactivation and mutations of p53 and PTEN tumor suppressors, and aberrant elevations of AKT and androgen receptor (AR) oncogenes lead to development and progression of PCa [26,27,28,29,30]. Ubiquitination, together with other PTM machineries, effectively controls protein levels of these factors to regulate cell function. For example, the compartmental localization, transcriptional activities and levels of p53 proteins in cells are mediated by MDM2, the major E3 Ub ligase of p53, through the ubiquitination pathway. Moreover, MDM2 elevation with p53 loss/mutation is found in cancers. p53 proteins can also be targeted by other E3 Ub ligases such as CHIP and Cul5 [31], and ARF stabilizes the p53 protein by antagonizing the MDM2-P53 interaction. ARF inactivation suppresses the growth of PCa cells and prostate tumorigenesis [32], and ARF ubiquitination is regulated by the E3 Ub ligase for ARF (ULF) through N-terminal polyubiquitination in a lysine-independent manner [33,34]. The nuclear localization and stability of PTEN protein are controlled by NEDD4-1, a HECT-domain E3 Ub ligase, through a mono-ubiquitination and poly-ubiquitination procedure, respectively [35,36]. Subtle variations and point mutations of the PTEN protein contribute to the incidence and progression of cancers through dysregulation of multiple oncogenic signaling pathways including PI3K/mTOR. Additionally, studies showed that PTEN ubiquitination is more complicated than previously expected as NEDD4-1 has been reported to be a downstream target of the PTEN-mTOR pathway [37]. The stability of the PTEN protein is also mediated by CHIP E3 Ub ligase and the phosphatase activity of PTEN is modulated by RFP-mediated ubiquitination [38,39]. The NEDD4-1 oncoprotein is downregulated by SCF β-TrCP E3 Ub ligase-mediated degradation when phosphorylated by Casein Kinase-I (CKI) [40]. Interestingly, the SCF β-TrCP E3 Ub ligase displays both oncogenic and tumor suppressive effects on PCa, depending on the triggered cascades of target proteins and oncogenic contexts [40,41,42]. Together, these lines of evidence highlight the notion that the functions and levels of p53 and PTEN proteins may be regulated through ubiquitination by several E3 Ub ligases under various stimuli. Upon PTEN inactivation and PI3K overexpression, hyper-activation of AKT produces a potent oncogenic impact on prostate tumorigenesis. AKT signaling and its membrane recruitment are ubiquitination-dependent cascades mediated by TRAF6 and NEDD4-1 E3 Ub ligases [43,44].

AR is a steroid hormone receptor and its aberrant elevation drives the development of PCa by upregulating downstream targets and co-factors in cell proliferation. The levels and activities of AR protein are controlled by several E3 Ub ligases through ubiquitination for protein degradation (CHIP, MDM2, SKP2) or the augmentation of transcriptional activity (RNF6, Siah2) [29,45,46]. RNF6 E3 Ub ligase predominantly ubiquitinates AR at lysine residue 845 to promote transcriptional activity [47], while SKP2 E3 Ub ligase mainly targets through lysine residue 847 of AR for proteasomal degradation [29]. It is possible that these E3 Ub ligases synergistically mediate AR signaling for PCa progression, but these studies have certainly provided some valuable information on the selection and application of ubiquitination-targeted drugs. SKP2 is an E3 Ub ligase for cell-cycle regulators and tumor suppressors such as CDK2, p27, p21 and DAB2IP in PCa cells [48]. SKP2 regulates AKT signaling indirectly by decreasing the ErbB-receptor-mediated AKT ubiquitination [28], and affects AR signaling directly by reducing AR ubiquitination to produce a detectable level of AR protein in PC3 and DU145 PCa cells [29]. SKP2 inactivation leads to a significant inhibition of cell proliferation *in vitro* and a suppression of prostate tumorigenesis *in vivo*. However it has been observed that SKP2 deficiency also contributes to dysregulation of other signaling pathways in PCa cells that in turn may antagonize the suppressive effects. Concomitant inactivation of *Pten* and *p53* genes leads to a highly aggressive phenotype of PCa in mice, and *Skp2* deficiency partially reduces, but does not completely block prostate tumorigenesis in *Pten*/*p53* mutant mice [49,50]. Further studies revealed that SKP2 inactivation contributes to the elevation of Jumonji/ARID1 B (JARID1B) (also named as KDM5B or PLU1) protein through TRAF6-mediated ubiquitination in cultured PCa cells and prostate tumors of mice. JARID1B upregulates AR but acts as a histone demethylase of histone 3 lysine 4 tri(di)methylation (H3K4me3/2) in PCa [51], implicating a connection between SKP2 E3 Ub ligase-mediated ubiquitination and histone modifications. These studies suggest a novel strategy in which a combined inhibition of SKP2 and AR (or PI3K/AKT and AR) pathways may improve the efficacy for treatment of PCa including castration-resistance PCa (CRPC), particularly in the context of PTEN loss. Recent studies have proposed that the ubiquitin landscape (termed the ubiquitylome) upon genetic alterations of oncoproteins determines prostate tumorigenesis [52]. The authors discovered that cancer-associated mutations of the cullin-RING Ub ligase adaptor protein speckle-type POZ protein (SPOP) cause an ubiquitylome shift to promote PCa. Because of the critical role of ubiquitination pathway on the activation of both AR and AKT, an ubiquitylome mapping may provide some clues on mechanisms of the reciprocal regulation of PI3K/AKT and AR signaling in PTEN-deficient cancers [53]. Given the indispensible roles of the ubiquitination pathway in human diseases and cancers, the E3 Ub ligases and associated co-factors have been recognized as potential targets for treatment of diseases and cancers.

Compared to studies on E3 Ub ligases in ubiquitination, advances in deubiquitination and the Ub proteasome system (UPS) are less documented, particularly for their functional roles in cancers. Several studies demonstrated that dysregulation of deubiquitination processes cause aberrant alterations of signaling cascades and cellular functions, which may accelerate the development of diseases and cancers. For example, overexpression of USP7 deubiquitinase promotes hepatocellular carcinoma (HCC) progression [54], and USP2a overexpression is detected in aggressive human PCa specimens [55]. Overexpression of CYLD, a DUB for K63-linked ubiquitination of AKT, decreases AKT activity mediated by TRAF6 [56]. Conversely, CYLD knockdown promotes cell proliferation and tumor growth of PCa [14].

### 2.2. SUMOylation in Prostate Cancer

Aberrant regulation of multiple signaling pathways including PTMs governs the initiation and progression of PCa [57,58,59,60]. The SUMOylation pathway, as one of the important PTMs, plays critical roles in various cancers including PCa by mediating the oncogenic functions of some key oncogenes and tumor suppressors [18,61,62,63]. Importantly, increasing evidence revealed that dysregulation of the SUMOylation pathway itself can generate biological impacts on the development of male mice, underscoring its critical role on prostate morphology and PCa. The levels and localizations of SUMO-1 display distinct functions in heterochromatin organization in male sperm cells of testis [64]. The elevation of UBC9, the SUMO-1 conjugating enzyme (E2), promotes cancer cell proliferation, tumor growth and metastasis [65]. UBC9 is involved in the transcriptional activity of AR target genes by SUMOylation of AR co-factors [66], and its levels are elevated in primary PCa but are decreased in metastatic PCa as compared to normal tissues [67]. By contrast, UBC9 inactivation causes defects in nuclear organization, which likely results in early embryonic lethality in mice [68]. PIASx, a SUMO E3 ligase and AR co-factor, is highly expressed in testis, and its deficiency results in small testis with a reduced number of sperm cells in mice [69]. Similarly, expression of alternative read frame (ARF) gene is required for spermatogenesis, and ARF inactivation also reduces sperm production in mice [70]. ARF contributes to the SUMOylation of early growth response (EGR1) transcription factor by forming the ARF/UBC9/SUMO-1 complex [71]. More studies reveal that ARF displays a novel and unique oncogenic function in PCa, and ARF levels are significantly correlated with the severity of advanced PCa specimens with PTEN loss [30,32]. ARF inactivation remarkably reduces prostate tumorigenesis of *Pten*/*p53* mutant mice by decreasing the stability and SUMOylation of SLUG protein [72].

It is well known that aberrant alterations of the PTEN/PI3K/AKT pathway plays a pivotal role on PCa, and that loss of the PTEN tumor suppressor drives the hyperactivation of PI3K/AKT/mTOR signaling to cause PCa [73]. Recent discoveries demonstrated that cellular functions and tumor suppressive roles of the PTEN protein are regulated through SUMOylation. The phosphatase activity of PTEN requires its SUMOylation at lysine 244 and lysine 266 mediated by SUMO-1 for its cytosolic localization, which contributes to an antagonizing effect on the activation of the PI3K/AKT pathway [74]. PTEN SUMOylation is catalyzed by PIASxα SUMO E3 ligase, leading to an increased stability of PTEN protein [75]. However, PTEN SUMOylation decreases its ubiquitination state, indicating a competitive correlation of SUMOylation and ubquitination on PTEN [75]. An independent study reported that PTEN can also be SUMOylated at a different site, lysine 254 of the PTEN protein, for nuclear localization, and SENP1 or SENP2 is likely to act as the SUMO-specific protease to reduce PTEN SUMOylation [76]. In agreement with this notion, SENP1 elevation is found in PCa specimens, and overexpression of SENP1 promotes the cell invasion, progression, metastasis and growth of CRPC [77]. These results indicate that SUMOylation contributes to both cytosolic and nuclear localizations of the PTEN protein at different lysine residues depending on growth stimuli or environments. Therefore, nuclear PTEN and its cancer-associated functions are controlled through both SUMOylation/deSUMOylation and ubiquitination/deubiquitination pathways [36,74,76,78]. Furthermore, AKT activation for tumorigenesis depends on not only the polyubiquitination mediated by TRAF6 but also the SUMOylation mediated by PIAS1 and SUMO-1 [43,79,80]. The oncogenic activity of JARID1B/KDM5B/PLU1 in PCa is likely mediated by ubiquitination and SUMOylation machineries in a synchronic and competitive manner. Specifically, JARID1B ubiquitination by TRAF6 E3 Ub ligase shares the same lysine residue 242 with JARID1B SUMOylation by hPC2 E3 SUMO ligase [50,81]. In contrast to TRAF6-mediated ubiquitination for JARID1B stability, JARID1B protein in the SUMOylated state is targeted by RNF4, a SUMO-target Ub ligase, for proteasome-mediated degradation [81]. 

The AR signaling pathway makes essential contributions to the development of normal prostate and PCa progression through regulation of PTMs including SUMOylation [30,82,83]. The SUMOylation of the AR protein is primarily elongated at lysine residues 386 and 520 with SUMO-1 and SUMO-2/3 molecules through the catalytic conversion of PIAS1 and PIASxα SUMO E3 ligases [84,85,86]. AR forms a protein complex with SUMO-1 and UBC9 for SUMOylation [84]. Interestingly, AR SUMOylation results in a decrease of the transcriptional activity of AR, and SUMOylation-deficient mutations of AR at K386A and/or K520A increase the transcriptional activity of AR in an androgen-dependent manner [84]. However, SUMOylation of co-factors such as AKT, FOXA1, Pontin, and hZimp10 in AR signaling promotes the transcriptional activity of AR in PCa cells in a pathway-dependent manner [87,88,89,90]. FOXA1 protein is mainly expressed in prostate epithelial cells, and its interaction with AR facilitates PCa progression [91,92,93]. However several lines of evidence pinpoint that FOXA1 loss also contributes to PCa by mediating the chromatin binding through androgen response elements (AREs) [94,95]. Most recent studies report that the SUMOylation of FOXA1 protein at lysine residues 6, 267 and 389 affects its nuclear mobility to regulate transcriptional activity of AR [87].

SENP1 and SENP2 are reported to be the deSUMOylation enzymes that cleave the SUMO-1 group from SUMOylated AR protein [96], and the presence of the deSUMOylation process provides a safeguard machinery to maintain the low detectable levels of SUMOylated proteins in cells (only 5%–10% of substrate proteins). Enforced overexpression of SENP1 increases AR transcriptional activity, and SENP1 knockdown decreases the growth of LNCaP PCa cells by downregulation of AR target genes [96,97]. Furthermore, the levels of SENP1 protein are higher in LNCaP, DU145, and PC3 PCa cells as compared to that in non-tumorigenic RWPE-1 cells, and SENP1 protein levels are correlated with malignant severities (Gleason scores and pathological stages) in human PCa specimens [98]. Results from animal models further confirmed that overexpression of SENP1 in mouse prostate leads to the development of high-grade prostatic intraepithelial neoplasia (HGPIN) by modulating multiple signaling pathways such as hypoxia-inducible factor 1 (HIF1), AR and cyclin D1 [22,99]. Therefore, targeting SENP1 and associated pathways is likely to be a novel and promising chemotherapeutic strategy for PCa treatment.

In addition, SUMOylation is involved in the regulation of many other transcription factors and non-transcription factors for cellular functions in PCa malignancy [100]. One study reported that there are about 900 proteins modified with SUMO molecules in PCa cells with the application of high-throughput screening and bioinformatic analysis [101]. As reported, SUMOylation modification regulates cellular functions of several well-known proteins including MDM2, p53, NF-κB, GATA, and SLUG [72,100]. For example, SUMOylation of p53 enhances its transcriptional activity to induce cellular senescence that acts as an anticancer mechanism in PCa [27,102], yet the activation of the cellular senescence program also requires expression of the E3 SUMO ligase PIASy [103]. On the other hand, the level of p53 SUMOylation is positively regulated by the presence of MDM2 and ARF [104]. ARF mediates the SUMOylation of many proteins including MDM2 and SLUG [72,105]. PML SUMOylation is essential for the formation of PML nuclear bodies [106,107], and PML loss accelerates Pten prostate tumorigenesis by regulation of nuclear AKT [108]. NF-κB signaling is aberrantly elevated in PCa [109], and NF-κB SUMOylation mediated by SUMO-2 decreases its activity [110]. Most recently, SUMOylation is found to play an important role on cell reprogramming by regulating expression and functions of several stem cell markers such as Oct4, Sox2 and Nanog [111,112]. SUMOylation of Oct4 increases its stability and transactivation to upregulate Nanog, while SUMOylation of Nanog and Sox2 results in a decrease of Nanog expression [112]. Meanwhile, Nanog is also regulated by the nuclear form of MET in PCa during cell reprogramming [49], yet it remains to be defined whether MET is SUMOylated in the nucleus of PCa cells.

## 3. Ubiquitination and SUMOylation in Diseases

### 3.1. Ubiquitination in Diseases

In addition to its potent roles in cancers, the ubiquitination pathway also plays important roles in human diseases including immune disorders, aging, diabetes and cardiomyopathies as it is involved in the regulation of some key signaling pathways (Figure 3) [3,113,114]. The canonical NF-κB pathway is essential for the development of immune responses as well as anticancer mechanisms, and its dysregulation contributes to pathological symptoms in the immune system [3]. The ubiquitination of the I-κB kinase complex (IKK) and NEMO activates the canonical NF-κB signaling and downstream cascades, which prevents severe defects in immune responses and multiple epidermal tissues. Mutations and deletions of I-κB kinase complex (IKK) and NEMO proteins cause severe defects in skin, teeth, nails and vision as well as the central nervous system. Failure extension of linear Ub chains upon NEMO mutation impairs the activation of canonical NF-κB signaling. Disruption and alterations of the NF-κB signaling pathway make cells more susceptible to TNF-mediated cell death and causes incontinentia pigmenti, an X-linked genetic disorder. The activation of NF-κB signaling is affected by heme-oxidized IRP2 Ub ligase-1 (HOIL-1) whose deficiency causes chronic autoinflammation, muscular amylopectinosis and susceptibility to bacterial infections [115]. Ubiquitinations of XIAP (encoding X-chromosome-linked IAP) and its adaptor proteins contribute to the activation of NOD2 and NF-κB signaling.

Dysregulation of ubiquitination is associated with the development and progression of neurodegenerative diseases such as Parkinson’s, Alzheimer’s and Huntington’s diseases [3]. The aberrant accumulation of protein aggregates (aggresomes or inclusion bodies) and polyglutamine repeats in these diseases are caused by aberrant ubiquitination. The aggregates or repeats of specific proteins cause damage to neuronal cells and affect the subcellular localizations of the protein inclusions to perturb homeostasis and functions. Parkinson’s disease patients have an aberrant accumulation of aggregated α-synuclein protein (also called Lewy bodies) in the mono- or di-ubiquitinated forms at lysine residues, indicating that the polyubiquitination pathway is unable to degrade α-synuclein through the proteasome. In addition, the ubiquitination pathway also affects the function of mitochondrial proteins and oxidative stresses in Parkinson’s disease. E3 Ub ligase Parkin (Park2) interacts with PTEN-induced putative kinase 1 (PINK1) to maintain the normal function of mitochondria, and Park2 mutations and the dissociation are found in Parkinson’s disease. Mitochondrial membrane proteins are ubiquitinated by Parkin, and the ubiquitination activity of Parkin depends on PINK1-mediated phosphorylation. Alzheimer’s disease, another neurodegenerative disorder, is mainly caused by the aberrant accumulation of β-amyloid peptides and tau proteins in neurons and synapses. TRAF6, an E3 Ub ligase, is involved in the shuttling and degradation of ubiquitinated tau proteins, and tau degradation is inhibited by hyperphosphorylation upon the elevation of β-amyloid peptides. Huntington’s disease is caused by the polyglutamine-repeated protein aggregates from aberrant ubiquitination of huntingtin protein by TRAF6.

The ubiquitination pathway contributes to senescence and aging by regulation of oxidative stress and proteolysis processes. A reduction of the Ub proteasome system (UPS) and autophagy is recognized as the biochemical feature of aging, which results in a decline of cellular function and an accumulation of damaged and aggregated proteins. Knockdown of UBA1, an Ub E1 enzyme, results in typical features of aging: impaired motion and decreased lifespan in adult Drosophila [116]. However, the activities of Ub E1 and Ub E2 enzymes decrease significantly upon calorie restriction (an age-delayed environment) in treated mice as compared to that in control mice [117]. The central machinery of UPS and autophagy is mainly controlled through the mammalian target of rapamycin (mTOR) that is regulated by insulin or IGF-1 receptor (IR), insulin receptor substrate (IRS), PTEN and AKT. The activities and cellular functions of these factors are selectively regulated through ubiquitination by different E3 Ub ligases such as MG53, Fbw8, NEDD4 and TRAF6. Forkhead box O (FoxO) transcription factors, such as FoxO3 and FoxO6, are involved in the autophagy-lysosome pathway to remove damaged proteins. FoxO family proteins are ubiquitinated and regulated by different E3 ligases such as CHIP, MDM2, NEDD4 and SKP2 to execute their transcriptional functions associated with senescence and aging [118].

### 3.2. SUMOylation in Diseases

The SUMOylation pathway plays essential roles in human diseases through regulation of genome stability, gene expression, protein-protein interactions and nuclear functions [20,21,119]. Given that the SUMOylation of substrate proteins and co-factors may have consequential effects on the ubiquitination process, its contributions to diseases are closely associated with the symptoms caused by ubquitination. In many cases, SUMOylation and ubiquitination share the consensus or lysine residues in substrate proteins, which result in an antagonizing effect on PTMs. Similar to protein ubiquitination, dysregulation of SUMOylation is associated with the severity of neurodegenerative disorders. Aberrant activation of SUMO-1 contributes to Huntington’s disease by increasing the stability of polyglutamine-repeat protein aggregates in huntingtin protein [120]. SUMOylation of tau and α-synuclein proteins is detected in Parkinson’s and Alzheimer’s diseases [119]. In addition, recent studies revealed that SUMOylation also links to the development of cardiovascular disease and diabetes by regulating some key proteins such as Nrf2, lamin A, calcium-transporting protein SERCA2a and PPARγ [21,121]. The impaired balance of SUMOylation and deSUMOylation processes can affect the aberrant signaling pathways and cellular functions [122]. Deficiency of SUMO-1 or SUMO specific protease 1 (SENP1) causes heart disease by regulation of HIF1α [123,124,125].

## 4. Clinical Implications and Conclusions

Advances in mechanistic insights into both ubiquitination/deubiquitination and SUMOylation/deSUMOylation pathways have provided valuable information for the development of novel drugs to effectively combat cancers and other diseases. Tremendous progress has been achieved to successfully target these pathways for therapeutic purposes [3,10,126]. Bortezomib, an inhibitor of 26S proteasome-mediated ubiquitination, is approved by the FDA to treat multiple myeloma with possibilities for use in other solid tumors. Bortezomib can also suppress tumor growth of PCa by markedly inhibiting NF-kB ubiquitination [126]. Nutlin, a small molecule, disrupts the degradation of p53 protein mediated by MDM2 E3 Ub ligase through ubiquitination to induce senescence and to inhibit cell proliferation [3,27,126]. SZL-P1-41, an inhibitor of SKP2 E3 Ub ligase, suppresses the growth of PCa cells by induction of p27-dependent senescence [127]. Ginkgolic acid inhibits the formation of the E1-SUMO complex in the SUMOylation process to impair the activation of NOTCH1 in cancer cells [128,129]. One report showed that the activity of SUMO specific proteases (SENP) can be inhibited by SI2, a small molecule compound that significantly augments protein SUMOylation [101]. The anticancer efficacy of SI2 compound in PCa *in vitro* and *in vivo* deserves further investigation.

Overall, ubiquitination and SUMOylation as unique machineries of PTMs make important contributions to the initiation, development and progression of many human diseases and malignancy including PCa. Although present in only a small percentage of cells (less than 10%), target proteins modified with SUMO or Ub moieties in various linkages display dramatic impacts in the regulation of gene expression, genome instability, cellular functions, cellular senescence and stem cell reprogramming *etc.* In addition, the imbalanced procedures of ubiquitination/deubiquitination and SUMOylation/deSUMOylation can generate additional influence on dysregulation of multiple signaling pathways. Importantly, the ubiquitination and SUMOylation modifications of one target protein can be modulated differentially depending on stimuli or environments: (1) in a synchronized manner in which SUMOylation assists/determines the ubiquitin-mediated degradation [81]; (2) in a competitive manner in which SUMOylation reduces ubiquitination [89,112]; (3) or in an independent manner in which SUMOylation has little effect on ubiquitination [76,80]. However, there are still a great number of challenging questions on PTMs to be addressed in a disease-specific or a drug-resistant fashion to understand in-depth mechanisms and furthermore selections of specific inhibitors for therapeutic applications. Given the dynamic changes of ubiquitination and SUMOylation pathways at different stages of malignancy, the inhibition of ubiquitination or SUMOylation combined with other target-based options warrants further investigation in cancer treatment, including that for CRPC.
